# A Gene Wiki for Community Annotation of Gene Function

**DOI:** 10.1371/journal.pbio.0060175

**Published:** 2008-07-08

**Authors:** Jon W Huss, Camilo Orozco, James Goodale, Chunlei Wu, Serge Batalov, Tim J Vickers, Faramarz Valafar, Andrew I Su

## Abstract

This manuscript describes the creation of comprehensive gene wiki, seeded with data from public domain sources, which will enable and encourage community annotation of gene function.

Gene portals (e.g., Entrez Gene [1] and Ensembl [2]) and model organism databases (e.g., Mouse Genome Database [3], Rat Genome Database [4], FlyBase [5]) are popular and useful tools for researching gene annotation and enforcing data standards. These databases provide a large volume and diversity of information on each gene, including protein and transcript sequences, genome location, genomic structure, aliases, links to literature, and gene function. These sites are considered to be the definitive sources for these types of gene annotation. However, by their very nature as authoritative annotation sources, the data displayed on these sites must be subjected to a high degree of oversight by expert curators. In short, the data model used by gene portals and model organism databases focuses on large contributions from a relatively small number of contributors.

In contrast, the online encyclopedia Wikipedia uses a different model for collaboratively synthesizing knowledge, commonly referred to as the “Long Tail” [6]. Originally coined in reference to the power law relationship observed in Internet commerce, the Long Tail is typified by Wikipedia's relatively open data model that targets small contributions from a large population of contributors. Articles in Wikipedia can be freely edited by all users, including anonymous editors, and any registered user can create new articles. Established in 2001, the English Wikipedia currently contains over two million articles edited by over six million user accounts. A recent study found that the number of contributions from new editors (less than 100 total edits) in total equals the number of contributions from the most established editors (greater than 10,000 edits) [7], illustrating the collective importance of the Long Tail. Equally importantly, previous studies have shown that Wikipedia content on scientific topics rivals the online Encyclopedia Britannica in accuracy [8].

Despite the widespread use of Wikipedia for general interest topics, its use for scholarly subjects has been uneven. The potential power of applying the Long Tail model to gene annotation has been previously noted [9–11]. A loose organization of Wikipedia editors has spearheaded the creation and expansion of several thousand articles related to molecular and cellular biology (the “MCB Wikiproject”), including many gene-specific pages. These articles vary widely in quality, format, and completeness, ranging from relatively complete encyclopedic entries (e.g., “enzyme,” “oxidative phosphorylation,” and “RNA interference”) to very short collections of information called “stubs” (e.g., “amphinase” and “glomus cell”). As an example of the collaborative writing process, the article on RNAi has been edited 708 times by 232 unique editors since its initial creation in October 2002. On the subject of human genes, generally only the most well-characterized of genes and proteins have highly developed entries (e.g., “HSP90” and “NF- B”).

In principle, a comprehensive gene wiki could have naturally evolved out of the existing Wikipedia framework, and as described above, the beginnings of this process were already underway. However, we hypothesized that growth could be greatly accelerated by systematic creation of gene page stubs, each of which would contain a basal level of gene annotation harvested from authoritative sources. Here we describe an effort to automatically create such a foundation for a comprehensive gene wiki. Moreover, we demonstrate that this effort has begun the positive-feedback loop between readers, contributors, and page utility, which will promote its long-term success.

## Laying the Foundation for a Comprehensive Gene Wiki

**Figure 1:**
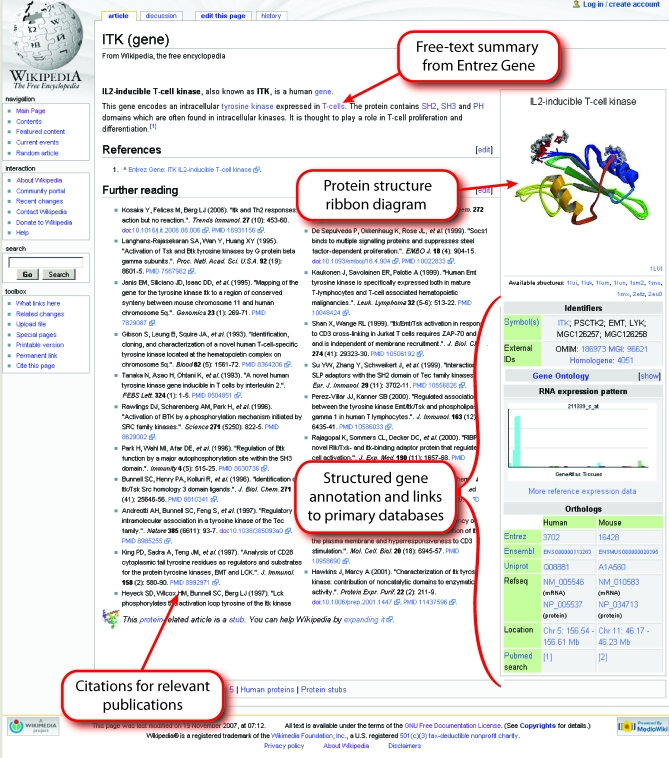
Example Gene Stub Created at Wikipedia for the ITK Gene All gene stubs include annotation harvested from public databases, including genome location, protein structures, anatomic gene expression patterns, and gene functions.

We first designed a gene stub with information based primarily on data from Entrez Gene (Figure 1). Each gene stub consists of a sidebar detailing the symbols and aliases, external identifiers, gene function (as represented in Gene Ontology), and genomic location. Although gene stubs are primarily focused on human genes, links to their mouse orthologs are also provided. When available, links to the Protein Data Bank are displayed under a thumbnail ribbon diagram, and gene expression patterns across diverse human tissues are shown as thumbnail bar charts [12]. Links to the primary databases are included when available. In addition, the central area of the gene stub shows a gene summary and a list of relevant references in the literature, both of which were provided by Entrez Gene.

After having finalized the content and format of the gene stubs with input from many Wikipedia users, we developed a computer program to generate gene stubs in automated fashion. (The program was developed using the Java programming language, and source code was released via the Apache License 2.0 at http://protein-box-bot.googlecode.com.) Gene annotation from the Entrez Gene database was downloaded for each gene, and properly formatted “wikitext” corresponding to the layout described above was created. The program then attempted to check if a gene page already existed. If not, the wikitext was automatically uploaded to Wikipedia, creating a new gene page titled according to the official gene symbol. If an existing gene page was detected, the wikitext was written to a log file for manual inspection and integration. All automatically generated content was added in templates that were made specifically in the context of this project so that subsequent updates of these data can be performed without disrupting manual edits made by other Wikipedia users. Gene pages were created in approximate order of decreasing number of references as indexed by Entrez Gene. As of February 2008, there were 7,500 new gene stubs created automatically and approximately 650 existing pages that were amended with content from our program.

## Evaluating Community Participation in Gene Annotation

**Figure 2:**
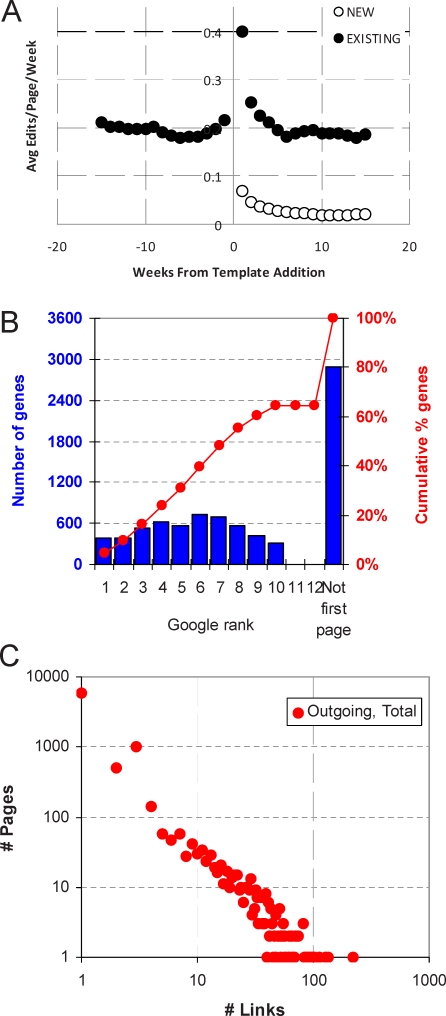
Analysis of Gene Wiki Impact (A) For the ~650 existing gene pages at Wikipedia that were amended with structured annotation, the cumulative rate of edits before and after addition was approximately the same. The rate of edits to the ~7,500 new gene pages was approximately 10-fold less than for the existing pages, though in total these edits accounted for approximately half of all edits to gene pages at Wikipedia. (B) When searching by gene symbol, Wikipedia gene pages are highly ranked by the Google search engine. Approximately 66% of all gene pages are found on the first page of search results. (C) The gene wiki can be analyzed in a network representation, where hyperlinks to other Wikipedia pages constitute network edges. Analysis of the network topology shows that connectivity follows a power law, indicative of a scale-free network.

To assess the effect of these gene stubs on Wikipedia, we first surveyed the edit logs for each of these pages that track contributions from the community (Figure 2A). For the 650 gene pages that were previously existing and amended in this effort, we found that the edit rates were roughly equal when comparing activity both before and after our automated efforts. Among the 7,500 new gene stubs created, edit rates were on average 10-fold less than for the pre-existing pages. However, due to the substantial difference in size of these groups, approximately 50% of all edits to gene pages were made on the newly created pages. These results demonstrate that in terms of the absolute number of edits, this effort roughly doubled the amount of mammalian gene annotation activity in Wikipedia. Given the relatively short period of time for which these entries have been available and the fact that this effort has not been previously announced in publication, we expect the rate of editing activity to continue to grow.

As another indication of the current and potential impact of these gene pages, we examined the ranking of these gene pages by the search engine Google. When searching by gene symbol, over 60% of gene pages at Wikipedia are listed in the first page of hits (Figure 2B). (Google ranks separated by pre-existing versus new pages are shown in Figure S1.) Previous research has shown that ranking in search results strongly influences users' navigation choices, and web users rarely navigate to lower-ranked search results [13]. Since any effort utilizing the Long Tail is dependent on a critical mass of users, the high visibility of these gene pages strongly suggests that the Wikipedia gene wiki will continue to grow, in the number of both readers and editors.

Finally, because Wikipedia entries allow for simple hyperlinking from gene pages to related concepts (as well as between two related gene pages), we examined the characteristics of the network topology surrounding these gene pages. Edges in the network denote hyperlinks, which can be further characterized by their directionality. When examining links that were directly referenced in the text (due to technical limitations we focused solely on outgoing links), we found that the network degree roughly followed a power law (with a slight excess of pages with low degree), indicative of a scale-free network (Figure 2C). (Network degree separated by pre-existing versus new pages is shown in Figure S2.) This scale-free property has been observed in many “real-world” networks, including the organization of the Internet [14] and of social networks [15]. In biology, scale-free networks have been observed in networks derived from protein interactions [16] and metabolic pathways [17,18].

The existence of scale-free properties in the Wikipedia gene network has at least two implications. First, scale-free networks are known to have a small network diameter (represented by the shortest path between any two concepts), suggesting that the Wikipedia gene network facilitates the efficient “browsing” of related topics in the local gene neighborhood. These related articles can link to other genes (linked by protein family or biological pathway, for example), as well as basic biological processes, disease states, specific researchers, and experimental methodologies. Second, the current scale-free property in gene wiki connectivity has potential implications for the gene wiki's ability to grow. We expect that gene wiki users will add links that reflect relationships between genes, including physical, functional, and regulatory interactions. As noted above, many of these types of biological networks are also thought to be scale-free. Because wiki pages have been shown to easily handle a large number of links (usually as inline hyperlinks) between related concepts, we expect the gene wiki to naturally accommodate the scale of relevant biological networks. In support of this hypothesis, recent analysis of the power law relationship in Wikipedia links [19] reveals a network having roughly the same degree distribution as seen in protein interaction networks [16].

As alluded to previously, the success of this gene wiki effort relies on a positive-feedback loop between page utility, the number of readers, and the number of editors. It is commonly recognized that new wiki editors are more likely to edit an existing page rather than to create a new one. Therefore, this stub-creation effort represents the critical first step in that positive feedback loop—the creation of useful initial wiki pages. As suggested in Figure 2B, these stubs will attract some reasonable population of readers, a small fraction of whom will additionally edit and improve the page. It is then hoped and expected that this positive-feedback loop will become self-sustaining.

## Leveraging the “Long Tail” for a Successful Gene Wiki

By leveraging the Long Tail of scientists in the annotation process, we believe that the gene wiki described here will harness a powerful and untapped source of gene annotation that is complementary to existing resources. Moreover, the type of data displayed by this gene wiki is also complementary in nature to other databases. Whereas existing gene portals are primarily focused on structured annotation data (ontologies, genome coordinates, etc.), the gene wiki is primarily focused on unstructured content in the form of free text, images, and diagrams that are more typical of review articles. Finally, the boundless nature of Wikipedia also allows for a single source to be appropriate to multiple levels of readers, from a lay audience to students to working professionals and academics.

However, the Long Tail and Wikipedia also have potential liabilities. Most notably, the completely open and anonymous nature of Wikipedia raises potential concerns about the completeness and accuracy of the articles, and in turn, the potential to recruit the broader scientific community to participate. We believe that the success of a gene wiki relies on the same foundation underlying the success of the rest of Wikipedia. Specifically, a simple editing syntax and a detailed version history for all pages enable effective collaboration and quick correction of incorrect or misleading content. A sizable population of readers then serves simultaneously as consumers, reviewers, and editors of content. Wikipedia has also managed to maintain an effective culture of collaboration by adherence to and promotion of its five core pillars (included in which is a code of conduct and maintenance of a neutral point of view) [20]. Finally, editors tend to add pages of interest to their “watchlist,” which highlights further changes to the article by other editors. Regardless of the specific reasoning, it is difficult to contest Wikipedia's success thus far in the breadth and accuracy of articles [8].

In addition, viable alternatives to Wikipedia exist. For example, a parallel gene wiki effort at Citizendium (http://www.citizendium.org) is also being considered, which, among other differences, requires authors to use real names and provides an explicit role for expert editors [21]. Despite potential concerns, this gene wiki project was initiated at Wikipedia to take advantage of the existing critical mass of articles that can be linked to and from gene pages, and for the critical mass of editors who are skilled in other aspects of online collaboration (copy editing, dispute resolution, governance, etc.) Although several other gene wikis already exist, none currently has access to a large user base and favorable search engine rankings of Wikipedia.

Importantly, this gene wiki effort is not meant to be a substitute for existing resources. Gene portals and model organism databases will continue to serve as authoritative references with a specific role for data curation and enforcement of data standards. Moreover, the structured and typed data in gene portals is amenable to incorporation into pipelines and systematic analyses in a way the information in a gene wiki cannot [22]. Most importantly, because articles are dynamic and not subject to rigorous peer review, the gene wiki is not intended to be a reference that is cited in a traditional peer-reviewed article or used exclusively as a source of gene annotation. Nevertheless, we believe that this gene wiki will be a valuable launch pad for collaboratively summarizing knowledge, and we expect that scientists will synergistically use the gene wiki with traditional gene portals.

Despite its infancy, the gene wiki effort has already had a substantial and growing impact on the Wikipedia community. We believe that this effort will encourage further contributions from scientists around the world and become a robust, cross-referenced tool for students, educators, and researchers everywhere. With the entire community's input, we envision this gene wiki evolving into a collection of collaboratively created, continually updated, community-reviewed review articles for every gene in the human genome.

## Supporting Information

Figure S1 Analysis of Google Rank as Stratified by New Versus Pre-Existing Wiki Pages These data are presented as described in Figure 2B.
(40 KB PPT).

Figure S2 Analysis of the Outgoing Network Degree as Stratified by New Versus Pre-Existing Wiki Pages These data are presented as described in Figure 2C.
(38 KB PPT).

## Note Added in Proof

Recently, Mons et al. [23] described another effort that uses community intelligence, called WikiProteins.
